# Lasers, liposuction, and the results conundrum

**DOI:** 10.4103/0970-0358.41102

**Published:** 2008

**Authors:** Mukund Thatte

**Affiliations:** Editor, Indian Journal of Plastic Surgery, 402 Vimal Smruti, 770 Dr. Ghanti Road, Dadar, Mumbai - 400 014, Maharashtra, India. E-mail: mthatte@vsnl.com

Amongst all the super specialities born out of ‘General Surgery’, ours has perhaps metamorphosed the most. We started with tube pedicles to remake body parts shattered by the new explosives of the First World War. In fact, it was a unique combination of three things that led to this event. There was better steel to form better armour for soldiers, there were better chemicals to create more powerful explosives to shatter that steel, and finally there was the automobile to rapidly transport the injured soldier to a safe base hospital to meet people like Gillies, Converse, and Filatov - I beg forgiveness for ignorance regarding the historical figures on the German side. You therefore had clinical problems of tissue loss, which hitherto had resulted in death and therefore not really exercised the minds of surgeons. Those who were evolving into ‘Plastic Surgeons’ solved the problem and went on to get to the bottom of many more, giving birth to a super specialty whose forte has been to resolve the difficult issues. Inevitably, as issues were sorted out, the solutions have been adopted by ‘regional’ specialists. Plastic surgery has responded by finding yet newer areas to unravel. Tissue transfer with microsurgery, brachial plexus repair, and cleft craft are some of the areas that come to mind offhand. Under the name of aesthetic surgery, we now want to ‘crack’ aging. We may or may not succeed, but the attempt is on in full vigor.

That brings me to the crux of this piece. Of late on various forums, we hear of lasers removing blemishes, which were thought to be untreatable, of liposuction being able to remove 15 kg in one go - promising to transform a middle-aged 75-kg lady into a svelte and slim 60-kg person in one sitting. Then there are the thread lifts, which will keep the face lift at bay for a few years and may become an office procedure. Much has been said for and against it. I am amazed at these feats and acknowledge that once widely adopted, this will transform aesthetic surgery as we know it today. My grouse is about the evidence or lack thereof. Much of the claim is anecdotal and based on personal accounts exchanged during the break hour in conferences. Admittedly, people have presented work in meetings as well. What I rue is the lack of a large volume of peer-reviewed papers putting forth the claim with long-term results, including the triumphs and the tribulations. The success rate, the complications and above all the safety record. I want to sincerely encourage the practitioners of these techniques to gather their data and sit down and write. Unless we present new evidence in this fashion, the safety of these procedures remains under a cloud. In an increasingly litiginous world, the published paper by a peer is very useful evidence to justify doing any procedure without being labeled unethical and or dangerous.

I believe the data is out there, what is now needed is the will to write.

